# Preeclampsia and syncytiotrophoblast membrane extracellular vesicles (STB-EVs)

**DOI:** 10.1042/CS20220149

**Published:** 2022-12-13

**Authors:** Toluwalase Awoyemi, Ana Sofia Cerdeira, Wei Zhang, Shuhan Jiang, Maryam Rahbar, Prassana Logenthiran, Christopher Redman, Manu Vatish

**Affiliations:** Nuffield Department of Women’s and Reproductive Health, University of Oxford, Oxford, U.K.

**Keywords:** Extracellular vesicles, nanoparticles, Placenta, Preeclampsia, STB-EVs, syncytiotrophoblast

## Abstract

Preeclampsia (PE) is a hypertensive complication of pregnancy that affects 2–8% of women worldwide and is one of the leading causes of maternal deaths and premature birth. PE can occur early in pregnancy (<34 weeks gestation) or late in pregnancy (>34 weeks gestation). Whilst the placenta is clearly implicated in early onset PE (EOPE), late onset PE (LOPE) is less clear with some believing the disease is entirely maternal whilst others believe that there is an interplay between maternal systems and the placenta. In both types of PE, the syncytiotrophoblast (STB), the layer of the placenta in direct contact with maternal blood, is stressed. In EOPE, the STB is oxidatively stressed in early pregnancy (leading to PE later in gestation- the two-stage model) whilst in LOPE the STB is stressed because of villous overcrowding and senescence later in pregnancy. It is this stress that perturbs maternal systems leading to the clinical manifestations of PE. Whilst some of the molecular species driving this stress have been identified, none completely explain the multisystem nature of PE. Syncytiotrophoblast membrane vesicles (STB-EVs) are a potential contributor to this multisystem disorder. STB-EVs are released into the maternal circulation in increasing amounts with advancing gestational age, and this release is further exacerbated with stress. There are good *in vitro* evidence that STB-EVs are taken up by macrophages and liver cells with additional evidence supporting endothelial cell uptake. STB-EV targeting remains in the early stages of discovery.

In this review, we highlight the role of STB-EVs in PE. In relation to current research, we discuss different protocols for *ex vivo* isolation of STB-EVs, as well as specific issues involving tissue preparation, isolation (some of which may be unique to STB-EVs), and methods for their analysis. We suggest potential solutions for these challenges.

## Introduction

Preeclampsia (PE) is a multisystem disease of pregnancy characterized by new-onset hypertension and proteinuria and/or evidence of maternal acute kidney injury, liver dysfunction, neurological features, haemolysis, or thrombocytopenia, and/or foetal growth restriction and in severe cases, death [1]. It has a prevalence of 2–8% globally and poses both short- and long-term morbidity and mortality risks to both the mother (cardiovascular disease) and the foetus (prematurity, foetal growth restriction) [2,3]. PE can manifest as either early onset (<34 weeks, EOPE) or late onset (>34 weeks, LOPE). EOPE has effects on mother and foetus whilst LOPE tends to affect the mother more. In two previous reviews [4,5], we showed that syncytiotrophoblast (STB) stress underlies both EOPE and LOPE. This STB stress is ascribed to an oxidatively stressed placenta from (1) malplacentation and unremodelled spiral arteries in EOPE or (2) a compressed and senescent term or post-term placenta in LOPE [4,6]. The stressed STB releases multiple signals into the maternal circulation, which cause a systemic vascular response, endothelial dysfunction, and the diverse features of preeclampsia.

STB stress is identified microscopically by syncytial breaks, apoptosis, autophagy, necrosis, complement deposition, oxidative and organellar stress involving the endoplasmic reticulum and mitochondria all of which are not visible using conventional histopathological techniques [7,8]. The STB response to this stress can be classified as increased production of positive stress molecules such as soluble FMS-like tyrosine kinase 1 [9], (3) or reduced production of negative stress molecules such as placental growth factor [10] Other positive responses include increased release of syncytiotrophoblast membrane extracellular vesicles (STB-EVs) [11] and of multinucleated syncytial vesicles. The latter originate from STB syncytial knots, which are aggregates of old inactive syncytial nuclei with no evidence of apoptosis and which increase with gestational age and are induced by hypoxia [12]

STB-EVs contain various biomolecules such as proteins, messenger RNA, microRNA, transfer RNA and even DNA in their cargo [13]. STB-EVs are in themselves complex structures believed to target maternal organs and deliver complex messages [14–16]. They have been shown to be taken into macrophages, hepatic cells and to some extent endothelial cells causing the recipient cells to adjust their behaviour and their responses [14–16]. STB-EVs may represent a mechanism by which the placenta orchestrates maternal responses in normal pregnancy and in PE. Because increased release of STB-EVs is an integral part of the STB stress response, their biogenesis ties well with the two-stage model of PE [4].

In this review, we explore the potential use of STB-EVs to diagnose, predict, and monitor PE in pregnancy, the ability of PE STB-EVs to target multiple maternal organs, thereby translating local placental stress into systemic maternal stress and the potential mechanistic roles of STB-EVs in PE. Finally, we highlight some of the challenges faced by the STB-EV community.

## Classification of syncytiotrophoblast membrane extracellular vesicle (STB-EVs)

STB-EVs like all EVs can be classified according to their biogenesis as exosomes, microvesicles or apoptotic bodies [17].

Microvesicles are a larger subtype of EVs, 100–1000 nm in size, produced by budding of the cell membrane in response to cell activation and stress. Exosomes are smaller EVs (30–100 nm) secreted from multivesicular bodies [18]. Apoptotic bodies (1–5 μm) are a subset of apoptotic extracellular vesicles [19] which also include smaller ApoEVs, including apoptotic microvesicles (ApoMVs 0.1–1 μm) [20] and exosome-like apoptotic vesicles (<150 nm) [21]. Apoptotic bodies are formed by apoptotic disassembly, including plasma membrane blebbing, apoptotic membrane protrusion formation and fragmentation [22]. Apoptotic bodies are associated with cellular death and have not been analyzed in this review.

Recently, MISEV (minimum information for the study of extracellular vesicles) guidelines have suggested using characterization based on physical characteristics (e.g., size) until evidence of biogenic synthesis are provided [23]. However, the size cut-offs are not well defined and different labs use different techniques to perform vesicle sizing. We have defined STB-EVs as <220 nm (small) or ≥220 nm (medium and/or large).

STB-EVs have been described in the literature as placental debris [24], placental microparticles [25], placental EVs [26], STB microvilli, STB microparticles [27], STB microvesicles [28], STB microvillous membrane [29], STB-derived microparticles [30], and recently STB extracellular vesicles [31] (STB-EVs). This variation in vesicle definition has prompted us to summarize the terminology used in this review in Table 1. This lack of consistency in nomenclature is problematic for those working in the field. It may be more useful to use a unified terminology (e.g., STB-EV) with a description of the isolation techniques to allow data to be identified and compared and to use physical characteristic classification rather than biogenesis which is often not proven.

**Table 1:** Description of extracellular vesicle terminology used in this review and their definitions

Term	Abbreviation	Description
Syncytiotrophoblast membrane extracellular vesicles	STB-EVs	Extracellular vesicles of syncytiotrophoblast origin
Medium/Large syncytiotrophoblast membrane extracellular vesicles	Medium/Large STB-EVs	STB-EVs who are >220 nm in diameter
Small syncytiotrophoblast membrane extracellular vesicles	Small STB-EVs	STB-EVs whose size range <220 nm in diameter
Placenta perfusion derived syncytiotrophoblast membrane extracellular vesicles	pSTB-EVs	STB-EVs obtained from the physiologic model of *ex vivo* dual lobe placenta perfusion
Mechanical syncytiotrophoblast membrane extracellular vesicles	mSTB-EVs	STB-EVs obtained from mechanically agitated placenta tissue
In vitro explant culture derived syncytiotrophoblast membrane extracellular vesicles	eSTB-EVs	STB-EVs derived from placenta explant culture
Exosomes	EX	Extracellular vesicles typically 30–100 nm in diameter and produced through ESCRT (endosomal complex required for transport) dependent or independent endosomal processes
Microvesicles	MV	Extracellular vesicles typically 100–1000 nm in diameter and produced from budding of the plasma membrane
Apoptotic bodies		Membrane-enclosed vesicles (1–5 µm in diameter) produced during apoptosis be cellular fragmentation

## Biogenesis of syncytiotrophoblast membrane extracellular vesicles (STB-EVs)

There are two potential explanations for STB-EV generation. The main biogenic mechanism, the classical pathway results is the production of STB-EVs from activated syncytiotrophoblast cells [32]. This includes the production of small STB-EVs (including exosomes), medium/large STB-EVs (including microvesicles) or other extracellular vesicles.

**Figure 1:**
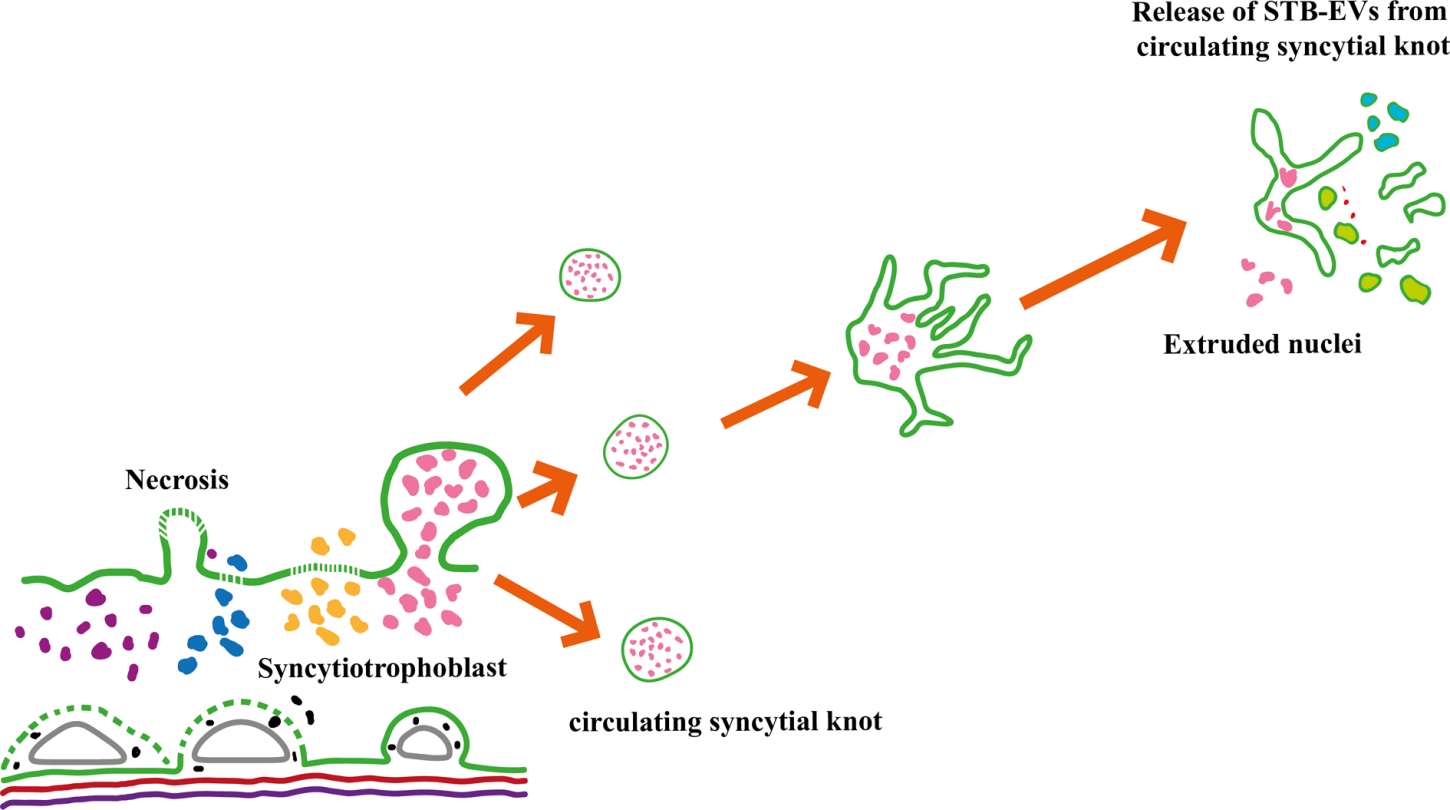
Schematic representation of the alternate STB-EV biogenesis theory proposed by Rajakumar et al. In preeclampsia, the syncytiotrophoblast is stressed leading to the increased release of multinucleated syncytial knots into the circulation. These circulating syncytial knots further fragment to produce different sized STB-EVs.

The less talked about pathway is the alternate pathway (Figure 1) proposed by Rajakumar et al., which involves shedding ‘small microparticles’ (EVs) from detached syncytial knots similar to the release of platelets from circulating megakaryocytes [33].

### Biogenesis of small STB-EVs (sSTB-EVs) including exosomes

No study has specifically examined the biogenesis of sSTB-EVs, but they are likely to behave comparably to other EVs. Hence, we speculate that sSTB-EVs (including exosomes which are 30–100 nm-sized extracellular vesicles) are generated through the ESCRT (Endosomal Complex Required for Transport) dependent or independent endosomal pathways.

**Figure 2:**
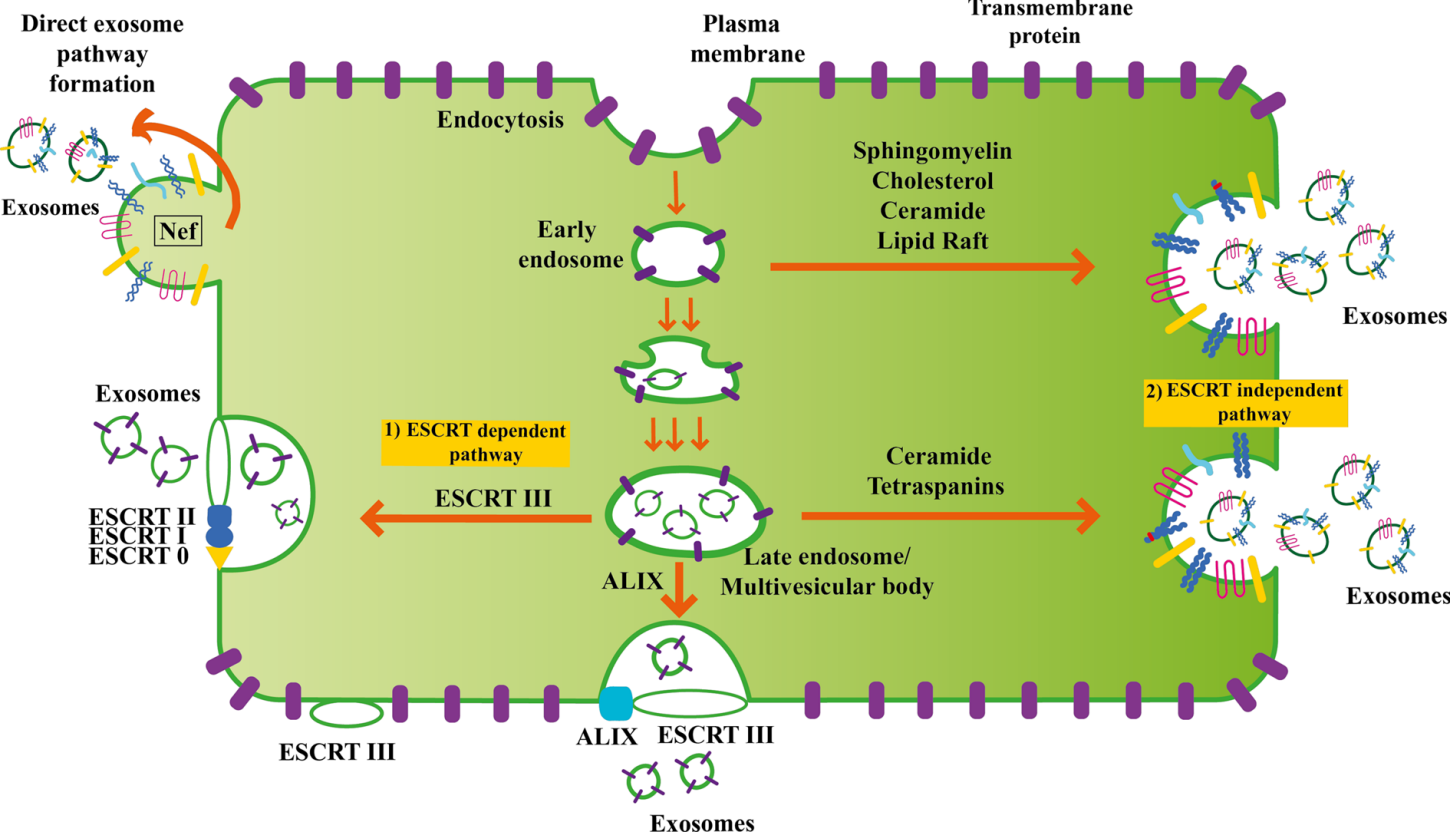
Biogenesis of exosomes through the ESCRT dependent and independent biogenesis pathways These biogenetic pathways involve several complicated processes facilitated by molecules such as tetraspanins, ceramides, sphingomyelin, cholesterol, ESCRT and ALIX.

In the ESCRT dependent pathway, intracellular early endosomes merge with, and deport their contents into, endocytic vesicles (Figure 2) for various downstream processes [17]. These endosome-endocytic vesicles can either be recycled (by fusing with lysosomes) [34] or can become late endosomes also called multivesicular bodies (MVB) or intraluminal vesicles (ILVs) [35]

MVBs are formed in two distinct processes, ESCRT dependent and ESCRT independent. The ESCRT dependent process itself involves two stages. The first involves the organization of the endosome membrane into specialized units highly enriched for tetraspanins (e.g., CD63-a marker of small STB-EVs [including exosomes]). The enriched tetraspanins facilitate interactions between themselves and either gangliosides [36], transmembrane/cytosolic proteins, or cholesterol [37] to form functional membrane microdomains. The second stage involves four ESCRT (Endosomal Sorting Complex Required for Transportation) multi-protein complexes [34]; ESCRT 0, I, II, and III. All the ESCRTs work in a stepwise manner. ESCRT 0 and ESCRT I subunits cluster ubiquitylated transmembrane cargoes on the microdomains of the external (limiting) membrane of MVBs and recruit ESCRT II and III subcomplexes. ESCRT I and II facilitate membrane budding. ESCRT III helps complete membrane budding via ALIX [17], which binds to the tumour susceptibility gene 101 (TSG101) component of the ESCRT I complex and the charged multivesicular body protein 4a (CHMP4) of ESCRT III [38]. ALIX also promotes exosome biogenesis through its interaction with syntenin [39].

In the ESCRT independent pathway (Figure 2), small STB-EVs/exosomes are produced through alternate pathways such as the ceramide pathway (requiring the generation of ceramide by neutral type II sphingomyelinase). Ceramide, like ESCRT, allows for the generation of membrane subdomains [40]. The tetraspanins, CD9, CD63, CD81 and CD82, are also involved in the ESCRT independent sorting mechanism based on the observation of defective exosomal biogenesis processes in tetraspanin animal knockout models [41,42]. ALIX and TSG101 are not involved in the ESCRT independent pathway and may thus be absent in this set of exosomes.

### Biogenesis of medium/large STB-EVs (m/lSTB-EVs) (including microvesicles)

**Figure 3:**
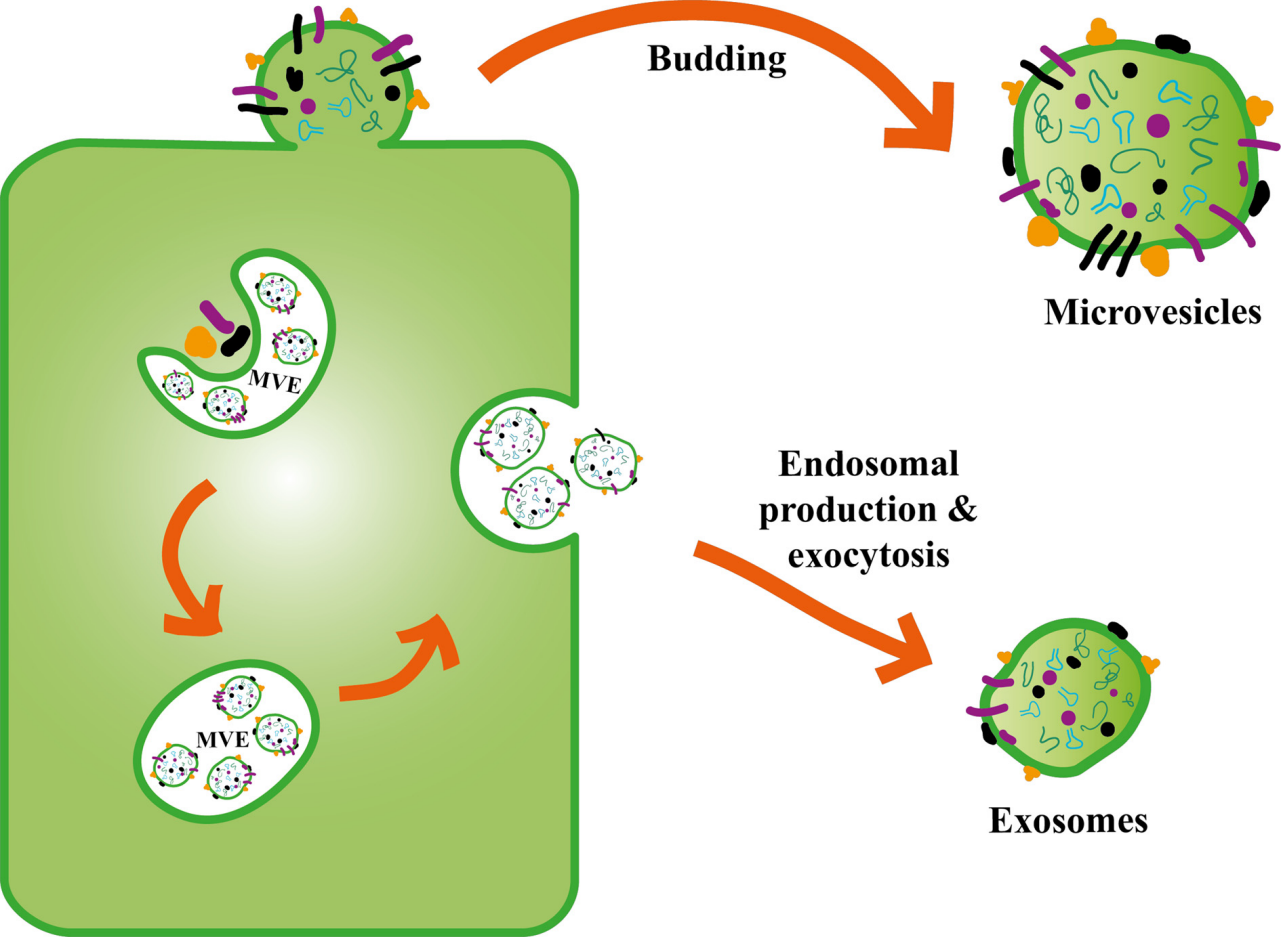
ESCRT (endosomal complex required for transport) dependent and independent exosome biogenesis pathways The ESCRT dependent pathway involves generation of extracellular vesicles via proteins such as ESCRT 0/I/II/III from late endosomal/multivesicular bodies (MVB) while the ESCRT independent pathway involves generation of extracellular vesicles from MVB by ceramide, cholesterol, spinghomyelin, lipid raft and tetraspanins.

In contrast with exosomes, microvesicle (m/lSTB-EVs) biogenesis is less well studied. However, m/lSTB-EVs are likely to behave comparably with other m/lEVs. Hence, we speculate that following a stressful stimulus (such as oxidative stress), calcium influxes into the STB leading to exposure and translocation of phosphatidylserine to the outer membrane leaflet by a cohort of enzymes; floppases, flippases and scramblases [41] (Figure 3). This translocation is followed by outward budding of the plasma membrane and contraction of cytoskeletal structures by actin–myosin interaction to deport the microvesicles (m/lSTB-EVs). Since PE is characterized by both oxidative and organelle stress, microvesicles are of relevance in PE and potentially more reflective of placental dysfunction in PE. The biogenesis of non STB microvesicles has been extensively reviewed by others [43].

## Isolation and enrichment of syncytiotrophoblast derived membrane extracellular vesicles

The process for isolating STB-EVs is highly variable (can be mechanically, perfusion or explant generated) as previously explained. The only comparative study that evaluated these three methods concluded that perfused STB-EVs and explant STB-EVs represented the most physiological method for the isolation of vesicles [27]. Ultimately, the goal of most STB-EV studies is to translate results to biofluids, which are hindered by a lack of consensus on the ideal blood sample type: plasma (EDTA or citrate) or serum. In 2013, the international society of thrombosis and haemostasis recommended citrated plasma as the plasma sample of choice, but this is not strictly adhered to by research labs for logistic and sample availability reasons [44]. The predominant use of serum in clinical circulating STB-EV studies is likely due to their large availability in biobanks compared to plasma samples. Serum may contain unwanted EVs released during clot formation and is perhaps less preferable to plasma samples. The anticoagulant type and downstream application are also a challenge regarding plasma. Citrated plasma (though the recommended anticoagulant) is less suitable than EDTA for (EV) RNA work, complicating matters further [45]. In addition to the challenges mentioned above, Coumans et al. have comprehensively identified other methodological challenges and considerations in studying extracellular vesicles in the circulation [45]. It would be of value to create a data repository for ‘omics studies (as has happened in oncology) to facilitate collaborative research, verification and translation of research experiments.

As described above, the two most physiological methods for syncytiotrophoblast derived EV isolation are (1) placental perfusion and (2) placenta explant culture [27].

### Perfused STB-EVs (pSTB-EVs)

Perfused STB-EVs (pSTB-EVs) are obtained through *ex vivo* dual lobe placenta perfusion (pSTBM). The foetal (flow rate of 6–12 ml/min and pressure of 50–90 mmHg) and maternal (flow rate of 10–20 ml/min and pressure of 80–140 mmHg) vessels of a suitable cotyledon/lobe of the placenta are cannulated and perfused with filtered modified M-199 medium with streptokinase (100,000 IU) [46]. The resulting maternal perfusate is then differentially centrifuged at 1500 ***g*** (15 min) to remove cellular debris, 10,000 ***g*** (30 min) for m/lSTB-EVs and 150,000 ***g*** (2 h) for sSTB-EVs.

### *In vitro* explant culture derived STB-EV (eSTB-EVS)

*In vitro* explant culture derived STB-EV (eSTB-EVS) involves isolating suitable villous tissue from the placenta, then cutting into thin strips (1–2 mm) and culturing in medium for 24–72 h to facilitate production of STB-EVs. A number of different differential centrifugation protocols have been developed. One example is centrifugation at 300 ***g*** (10 min), 2000 ***g*** (20 min) and 10,000 ***g*** (30 min) to remove cellular debris, and ultracentrifugation at 100,000 ***g*** (1.5 h at 4°C) to pellet small vesicles, including exosomes (sSTB-EVs) [47]; although other protocols have been successfully used.

p STB-EVs have a higher EV yield and are relatively cheaper to obtain than eSTB-EVs. Still, they require placenta perfusion knowledge and expertise and have a higher STB-EVs production failure rate (i.e., the proportion of unsuccessfully perfused placentas). It is also interesting that all STB-EVs (studies reviewed in this manuscript) have been enriched with differential ultracentrifugation (dUC) rather than other enrichment methods such as size exclusion chromatography (SEC), density gradient centrifugation (DEC), ultrafiltration, immunoprecipitation and immunoaffinity beads capture. This may be because of the familiarity of placenta EV researchers with differential ultracentrifugation (dUC), the popularity of differential ultracentrifugation, or the large number of source materials generated from the two most common STB-EV sources: placenta perfusion (as much as 600 ml) and placenta explant culture. The latter has the flexibility of scale up or down and allows monitoring STB-EVs in response to stimulation.

## Biodistribution of EVs and STB-EVs

Most data of EV biodistribution are from animal models due to challenges that currently lie with their study in humans, such as the safety of fluorescently labelled or radioisotope tagged EVs. Few animal studies have specifically examined pregnancy, but STB-EVs are likely to behave comparably to other EVs.

Non-pregnancy data show that EVs (especially small EVs) are rapidly cleared from the circulation within a few minutes by circulating immune cells [48]. Kang et al. extensively reviewed 38 studies and the time course of EVs in 20 different organs when administered into animal models [49]. They found that the liver was the principal organ of small EV localization with peaks as early as the first hour after administration which persisted for 3 days [49]. Intriguingly, the lungs also peaked in the first hour but diminished subsequently [49]. The kidneys showed low levels of small EVs in the first hour, followed by a gradual reduction in small EV levels, while the spleen showed moderate levels of small EVs from the 2nd till the 12th hour. To summarize, small EVs are mainly distributed in the liver and lungs. For the other organs studied, the localization was not as rapid. The bladder showed a peak at 4 h while the brain, gastrointestinal tract, and the heart gradually and slightly increased up to 24 h, and the skeletal system had moderate levels of EVs at 24 h [49].

Large EVs were less studied, but it has been shown that they rapidly localize to the lungs within 1 h, followed by a gradual decline over 12 h [49]. The large EVs gradually rose and peaked in the liver in the 24th hour. The kidneys had moderate levels of large EVs, while the brain, heart, and spleen had low to no levels of EVs in them 24 h post-injection [49]. In summary, the lungs are the main organs where large EVs are localized, followed by the liver.

In one of the few pregnancy biodistribution studies, Tong et al. evaluated pregnant mice. They noted that medium/large STB-EVs localized to the lungs after 30 min, and after 24 h they localized to the liver and lungs [50]. Oddly, they noted that the placenta EVs did not target the placenta [50]. They speculated that compared with the non-pregnant state, most STB-EVs are cleared by the kidneys and lungs rather than just the lungs (as in non-pregnant mice). They also concluded that this localization is unlikely to be due to size but rather due to ligand–receptor interaction, some of which have been described by Ngyuen et al., who observed that these STB-EVs are specifically trafficked to the pulmonary interstitial macrophages through outer membrane proteins and integrins (Integrins α3, αV, α5, β1, and β3) [51].

## Detection of STB-EVs in biofluids

STB-EVs have been found in the plasma (in normal, preeclampsia and gestational diabetes mellitus [GDM]) [52,53] and the urine (in normal and GDM but not in PE) [54]. STB-EVs carry placental alkaline phosphatase (PLAP) as a marker of STB origin. It was reported that (PLAP^+^) sSTB-EVs can be detected in the circulation as early as six weeks, increase with increasing gestation age, and peak at term [55,56]. They also circulate at a higher level in PE compared with normal. They reported that sSTB-EV levels fell rapidly postpartum (within 2 days), although this study purported to investigate EVs with very high PLAP levels which may have been large STB fragments [56,57]. m/lSTB-EVs have been reported in the circulation with elevated levels solely in the presence of EOPE and not in LOPE or gestation matched IUGR (intrauterine growth restriction) [58]. They demonstrated significant elevation of PLAP+ m/l STB-EVs above non-pregnant patients in all conditions. They noted no relationship between m/lSTB-EV concentration and parameters of clinical disease severity in the PE patients. These results seem at odds with the clinical observation that preeclampsia and eclampsia can occur up to 6-week postpartum. A potential mechanism for this clinical finding may be the presence of transcriptionally active placental material in the lungs, which is capable of producing pathogenic molecules such as sFLT-1 [33,59].

## Targeting and sorting of molecules into syncytiotrophoblast membrane extracellular vesicles

Identification of mechanisms that sort molecules into EVs is still largely unclear. No STB-EV-specific mechanisms have been studied, but general mechanisms of EV sorting are likely applicable.

Cytosolic proteins (such as heat shock [70 kDa] protein [HSP70]) are incorporated into vesicles with other proteins found ubiquitously on EVs, and glycosylphosphatidylinositol (GPI)-anchored membrane proteins are recruited into EVs because of their enrichment on lipid domains and rafts. ESCRT complexes sort transmembrane proteins that have been mono-ubiquitinated into intraluminal vesicles (ESCRT-dependent pathway) destined to be released as extracellular vesicles [60]. Mono- and poly-ubiquitinated proteins can also be sorted independent of ESCRT by sphingolipids, tetraspanins and ceramide [61].

Small RNAs (ribonucleic acid) such as microRNAs contain specific motifs which play a role in preferential exosome sorting [62]. Garcia-Martin et al. identified the presence of unique sequences/sorting motifs (EXOmotifs) enriched up to 80-fold in small EVs [62]. These motifs bind with two RNA-binding proteins, Alyref and Fus, to facilitate the sorting [62]. The KRAS-MEK signalling pathway as well as major vault protein, Y-box binding protein1, hnRNPA2B1, Caveolin-1, neural sphingomyelinase 2 (nSMase2) and argonaut 2 are also involved in miRNA sorting [63]. Groot et al. [63] have comprehensively reviewed the sorting mechanisms for microRNAs into extracellular vesicles, and an extensive review of general EV sorting mechanisms has been published by Anand et al. [64]. Little is known about sorting metabolites, lipids, and mRNA, tRNAs or other RNA subtypes asides from microRNA into EVs.

## Syncytiotrophoblast membrane extracellular vesicle cellular entry mechanisms

Extensive reviews of general EV cellular entry mechanisms have been done by others and a few of them have specifically evaluated STB-EV entry mechanisms.

Cronqvist et al. speculated that STB-EV uptake into primary human coronary artery endothelial cells might be mediated by endocytosis or membrane fusion [14]. At the same time, Vargas et al. observed that villous cytotrophoblast small STB-EVs are taken up by BeWo cells in a syncytin-1 and -2 dependent manner [14]. Li et al. showed that trophoblastic sEVs were transported into target cells via macropinocytic and clathrin-mediated endocytic pathways but not caveolin-dependent endocytosis [65]. We recently showed that hepatic cells can take up STB-EVs via apolipoprotein E, which is carried on a subset of these vesicles [15].

**Figure 4:**
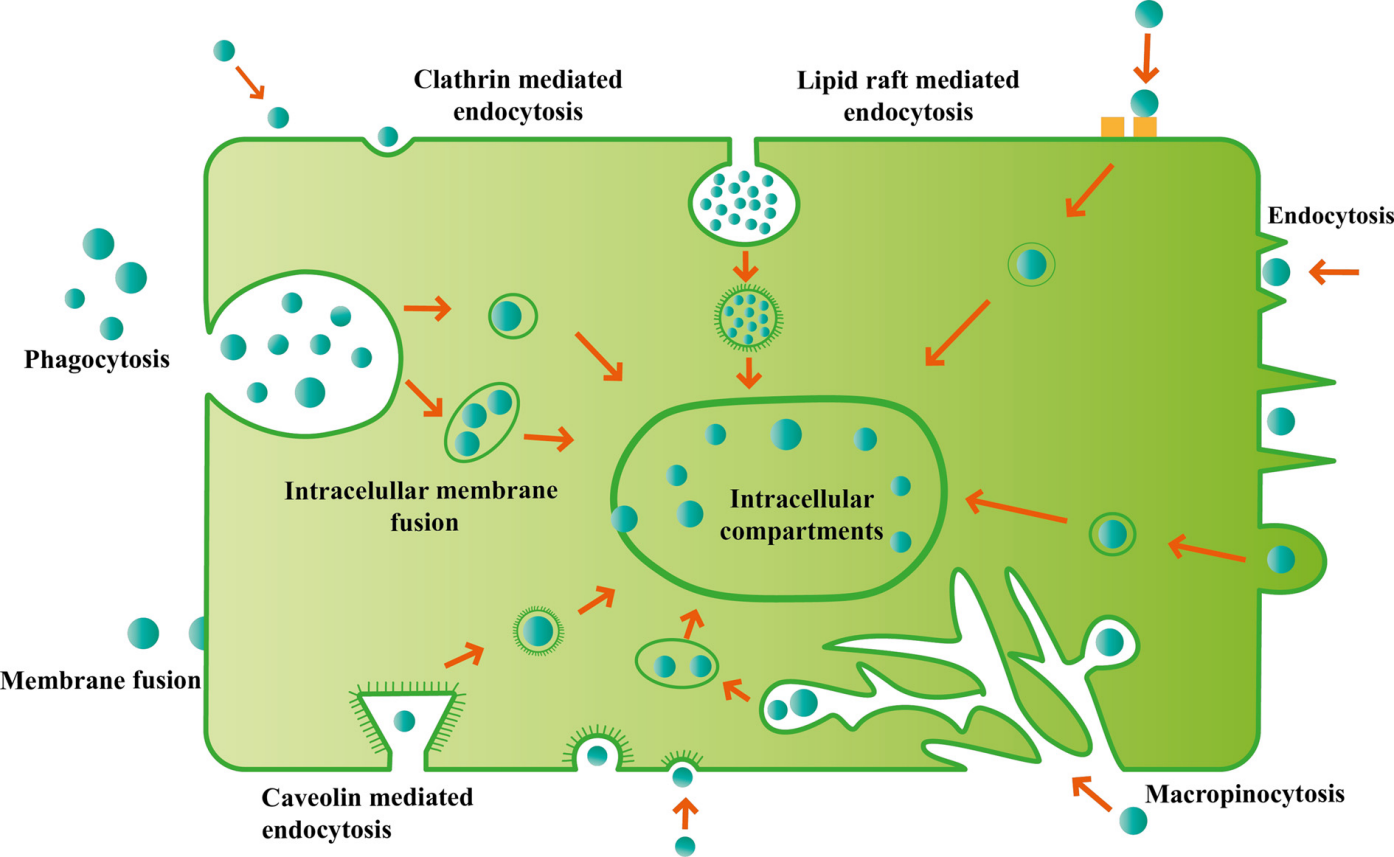
Extracellular vesicle cellular entry mechanisms EVs from donor cells are internalized by recipient cells via endocytosis, micropinocytosis, membrane fusion, phagocytosis or membrane fusion. Once internalized, these EVS are absorbed into the intracellular compartments and their contents deposited intracellularly.

It is likely that the mechanisms of general EV uptake by recipient cells (Figure 4) extends to STB-EVs. Since the discovery that EVs mediate downstream actions in recipient cells, intense research has been conducted to identify these mechanisms some of which include endocytosis [66], lipid raft mediated endocytosis, clathrin and caveolin-dependent endocytosis [67], RhoA-dependent endocytosis, phagocytosis [68], macropinocytosis [69] and membrane fusion [70]. Endocytosis, a cell’s extracellular material absorptive mechanism, occurs through heparan sulphate proteoglycans [71], galectin 5 [72], CD91 [73], calcium [74], fibronectin [75], or actin, depending on the cellular origin of the EV. Clathrin and caveolin-dependent endocytosis are mediated by dynamin [76] and neuroactive ligand receptors [67]. Lipid raft endocytosis (endocytosis that happens through specialised plasma membrane microdomains) involves glycosphingolipids [77], cholesterol [78] or ERK1/2 [79]. Macropinocytosis is mediated through ion pumps (proton pumps or sodium/proton exchangers) [76]. Phagocytosis occurs through phosphoinositide 3-kinases (PI3Ks) [80] or phosphatidylserine [76] while membrane fusion is through sodium reabsorption [70].

A comprehensive review of the general EV entry process has been previously reviewed by Mulcahy et al. [81], but no extensive study has been conducted to determine if the mechanisms of uptake of STB-EVs differ from other EVs or if uptake differs between medium, large and small (STB-) EVs. The complete characterization and profiling of surface markers carried on STB-EVs would facilitate the identification of STB-EVs specific entry mechanisms.

## Mechanisms of action of STB-EVs in preeclampsia

As discussed in the introduction, the STB-EVs in PE are a result of syncytiotrophoblast stress and are deported into the maternal circulation to target various maternal organs. Some of their effects on the hematologic and endothelial systems, both of which are implicated in the maternal syndrome have been partially characterized.

### PE STB-EVs are inflammatory

PE STB-EVs are proinflammatory, but this is true only in EOPE [82]. In studies where the phenotypes of STB-EVs are differentiated (i.e., EOPE vs LOPE), LOPE STB-EVs are less proinflammatory in THP1 cells compared with EOPE STB-EVs [16]. Trophoblast EVs have also been shown to carry multiple damage-associated molecular pattern (DAMP) molecules (e.g., HMGB1) in response to endoplasmic reticulum (ER) stress which can also activate the immune system [83]. STB-EVs showed an increase in proinflammatory cytokines (tumour necrosis factor [TNF]-α, IL-1, IL-8, IL-6) in neutrophils, B and T cells [84], monocytes [85], which can be inhibited by blocking Toll-like receptor (TLR) signalling or targeting the nuclear factor kappa-light-chain-enhancer of activated B cells (NF-κB) [86].

### PE STB-EVs are anti-angiogenic and cause endothelial dysfunction

Endothelial nitric oxide synthase (eNOS) activity is reduced in PE STB-EVs and may contribute to the vascular dysfunction and hypertension seen in PE [87]. In pregnant animal studies, injection of preeclamptic EVs results in decreased diameter of blood vessels, decreased maternal body weight, increased maternal blood pressure [88], and an increase in circulating sFLT-1 [89]. PE STB-EVs enhance cellular oxidative stress by inducing ER and mitochondrial membrane ruffling and can cause endothelial dysfunction by transferring functional microRNAs or mitochondrial DNA from dysfunctional syncytiotrophoblast [90]. They can bind proangiogenic factors (vascular endothelial growth factor [VEGF], placental growth factor [PLGF], and transforming growth factor β [TGF-β]), disrupt cultured human umbilical vein endothelial cell (HUVEC) monolayers [91] and inhibit endothelial tube formation *in vitro*, translating to antiangiogenesis [33].

In contrast, Brien et al. compared total-conditioned, EV-depleted, and EV-enriched media of PE and normal placental explants and reported that the soluble fraction induced leukocyte adhesion, endothelial dysfunction and significantly repressed angiogenesis in human umbilical cell endothelial cell (HUVEC) but not the EV-enriched fraction [92]. They concluded that soluble factors dominate the vascular pathology of PE not extracellular vesicles [92]. It is difficult to state why the results are conflicting, one factor might be the variation in endothelial cell models. However, it is undisputable that in-depth and extensive research are still needed to characterize the mechanistic role of STB-EVs in PE.

### PE STB-EVs promote abnormal coagulation

Tannetta et al. noted that PE STB-EVs could activate platelets by increasing protein tyrosine phosphorylation which can be halted with aspirin [46]. In addition, PE STB-EVs carry procoagulant proteins such as tissue factor (TF) [93], anionic phospholipids (PS) [94] and plasminogen activator inhibitors 1 (PAI-1) [95] which may contribute to the abnormal coagulation seen in PE.

Taken together, potential mechanisms of action recognised so far are largely attributed to proteins. Very little is known about the short- and long- term effects of STB-EV cargo contents in PE. It has been shown that microRNA from the C19 microcluster is almost exclusively expressed in placenta and exported within trophoblast EVs. These EV associated microRNAs can significantly alter cellular behavior such as conferring protection from viral infection by inducing autophagy as well as being trafficked from the maternal circulation to the placenta [96,97]. It would be interesting to speculate whether changes in microRNA content that occur in PE might further contribute to the clinical manifestations of the disease.

## Challenges of studying/researching STB-EVs in pregnancy and preeclampsia

The field of STB-EVs holds excellent promise as diagnostic, predictive or therapeutic agents in the management of PE. Most studies (reviewed in this study) use as universal syncytiotrophoblast markers either placenta alkaline phosphatase (PLAP) [98] or less often syncytin-1 or-2 [99,100]. Some PLAP antibodies may cross-react with other types of circulating alkaline phosphatases e.g., intestinal alkaline phosphatase when used in biofluids. Syncytin-1 does not discriminate between STB and CTB, but syncytin-2 is expressed only in STB (human protein atlas). However, syncytin-2 levels are reduced in circulating serum-derived PE exosomes [101] and consequently may not be as useful as a universal STB-EV marker. Isthmin-2 seems to be expressed predominantly in the placenta but is not exclusive to the syncytiotrophoblast.

The isolation and enrichment methods for STB-EVs is highly variable (can be cell or tissue-derived) as explained in the isolation of STB-EVs section, the only comparative study that evaluated some of these methods concluded that two methods; perfused STB-EVs and explant STB-EVs represented the most physiological method for the isolation of vesicles [102]. In addition, there is a lack of consensus on the ideal blood sample type; plasma (EDTA or citrate) or serum. In 2013, the international society of thrombosis and haemostasis recommended citrated plasma as the plasma sample of choice, but this is not strictly adhered to by research labs for logistic and sample availability reasons [44]. The predominant use of serum in clinical circulating STB-EV studies is likely due to their large availability in biobanks compared to plasma samples. Serum may contain unwanted EVs released during clot formation and is thus less preferred to plasma samples. The anticoagulant type and downstream application are also a challenge regarding plasma. Citrated plasma (though the recommended anticoagulant) is less suitable than EDTA for (EV) RNA work [45,103]. In addition to the challenges mentioned above, Coumans et al. have comprehensively identified other methodological challenges and considerations in studying extracellular vesicles in the circulation [45].

Undoubtedly, the resolution of these challenges and identifying an ideal marker would enable better STB-EV analysis in biofluids such as plasma or urine. It would also be helpful for the placenta EV community to create a data repository for large data studies to facilitate collaborative research, verification, and translation of research experiments.

## Diagnostic potential

### Diagnostic potential: STB-EVs contain potentially diagnostic microRNAs in preeclampsia

MicroRNAs are a sub-class of small non-coding RNAs of 19–24 nucleotides. MicroRNAs regulate the expression of mRNA by inducing mRNA cleavage or repressing translation. In the circulation, microRNAs can be carried by extracellular vesicles, argonaut 2 (AGO2) proteins, high-density lipoprotein, and other ribonucleoprotein complexes [104]. In PE, STB-EV microRNAs have been explored by investigating cell-free microRNA in the circulation (plasma or serum), identifying potential biomarkers, and linking them to the placenta. Srinivasan et al. summarized most of the previously identified PE extracellular microRNAs biomarker candidates such as has-miR-574-5p, has-miR-181, miR-122, miR-758, has-miR-517-5p, has-miR-520-5p, has-miR-521, has-miR-521, has-miR-520h, has-miR-542-3p, has-miR-4313, has-miR-23c, has-miR-191, miR-210 and miR-25 [105].

### Diagnostic potential: STB-EVs carry potentially diagnostic proteins in preeclampsia

Since discovering these EVs, investigations have been performed to identify protein differences on STB-EVs or placenta EVs in the context of PE. Gardiner et al. reported tissue factor (TF) to be up-regulated in PE STB-EVs (2 to10 fold increase) with increased TF activity compared with normal pregnancy.

Tanetta et al [106]. and Tong et al [107]. Noted FLT-1 to be up-regulated in PE STB-EVs with multi-colour flow cytometry and Western blot. FLT-1 is an antiangiogenic tyrosine kinase that is membrane-anchored and binds VEGF. Soluble FLT-1, the splice variant of FLT-1, has also been described as up-regulated in syncytial knots by Rajakumar et al. [33] Similar to FLT-1, soluble, vesicle- and syncytial nuclear aggregate-bound soluble FLT-1 bind freely circulating vascular endothelial factor (VEGF) and reduce the bioavailability of VEGF. This reduction in circulating VEGF leads to endothelial dysfunction, vasoconstriction and reduced endothelial nitric oxide production, all features of PE. Soluble FLT-1 is known to be increased in PE and further increased with severity [9]. Rajakumar et al. estimated that >25% of the measurable soluble FLT-1 in third-trimester maternal plasma are produced from syncytial knots [33].

Our group has previously reported biologically active neprilysin (NEP) as up-regulated in PE STB-EVs [108]. NEP is a metalloendopeptidase that inactivates bioactive peptides such as atrial natriuretic peptide (ANP), brain natriuretic peptide (BNP), endothelin, bradykinin and oxytocin. Similarly, transthyretin, a thyroxine and retinol-binding protein essential in foetal development during early pregnancy is up-regulated in placenta nanovesicles, including S STB-EVs [109,110]. Likewise, our group also previously reported the up-regulation of a novel glycosylated form of SIGLEC6, a subgroup of the negatively charged carbohydrate-binding lectins in the pericellular matrix of cells on PE STB-EVs [111]. Members of SIGLEC family are known to regulate cellular activation and clonal expansion of immune cells expressing SIGLEC ligand through cis- and trans-activation mechanisms, respectively [112]. Of note, SIGLEC6 is a high affinity receptor for leptin, STB-EV bound SIGLEC6 could potentially regulate bioavailability of leptin in the maternal circulation. The precise functions mediated by this glycosylated form of SIGLEC6 on STB-EVs are yet to be dissected.

Tersigni et al. observed human leukocyte antigen-DR isotype 3 (HLA-DR 3) to be present on 39% of PE medium/large STB-EV studied and none on NP medium/large STB-EVs. Motta-Mejia et al. found endothelial nitrogen oxide synthase (eNOS), an enzyme that synthesises the potent vasodilator, nitric oxide, to be down-regulated with reduced activity in PE STB-EVs compared with NP [87] Finally, placenta protein 13 (PP13) is down-regulated in PE STB-EVs compared with normal STB-EVs and may contribute to impaired tolerance and inflammation seen in PE [113].

### Diagnostic potential: STB-EVs carry potentially diagnostic lipids in preeclampsia

Baig et al. analysed the lipid content of m/lSTB-EVs (microvesicles) and reported significant up-regulation of phosphatidyl serine (PS) and down-regulation of phosphatidylinositol (PI), phosphatidic acid (PA) and ganglioside mannoside 3 (GM3) [114].

To our knowledge very few STB-EV studies have been conducted to identify differences in metabolites, messenger RNA, and DNA in PE STB-EVs compared with NP and would represent an important area of further study to help identify the roles of STB-EVs in PE.

## Conclusion

PE is a multisystem pregnancy disorder with diagnostic, mechanistic and therapeutic challenges. STB stress drives its pathology by stimulating release of STB-derived soluble factors and extracellular vesicles (STB-EVs), which are the putative propagators of the maternal systemic manifestations of the disease. PE STB-EVs are proinflammatory, anti-angiogenic and procoagulant, which are different facets of endothelial dysfunction, which in turn has been long known to be the cause of the maternal syndrome. Analysis of circulating STB-EVs exploits their potential as circulating liquid placental biopsies in PE, which may reflect the health of the syncytiotrophoblast and the foetus.

This review demonstrates the relevance of STB-EVs to the pathophysiology of PE, their potential uses in the diagnosis of PE, and the current challenges that confront researchers to achieve these ends.

## Data Availability

No data was used for this review, all referenced papers have been cited.

## Open Access

Open access for this article was enabled by the participation of University of Oxford in an all-inclusive* Read & Publish* agreement with Portland Press and the Biochemical Society under a transformative agreement with JISC.

## Abbreviations

ANP: atrial natriuretic peptide
BNP: brain natriuretic peptide
GDM: gestational diabetes mellitus
GM3: ganglioside mannoside 3
HLA-DR 3: human leukocyte antigen-DR isotype 3
HUVEC: human umbilical vein endothelial cell
PA: phosphatidic acid
PI: phosphatidylinositol
PLGF: placental growth factor
PS: phosphatidyl serine
STB-EV: syncytiotrophoblast membrane extracellular vesicle
TGF-β: transforming growth factor β
VEGF: vascular endothelial factor
